# Perspective on novel proteins encoded by circular RNAs in glioblastoma

**DOI:** 10.20892/j.issn.2095-3941.2021.0678

**Published:** 2022-02-14

**Authors:** Xixi Li, Xinya Gao, Nu Zhang

**Affiliations:** 1 Department of Neurosurgery, Sun Yat-sen University, The First Affiliated Hospital of Sun Yat-sen University, Guangzhou 510080, China

## Introduction

Circular RNAs (circRNAs) are generated by the non-canonical back-splicing of pre-mRNA and have been shown to be present in a wide variety of tissues at lower expression levels than their associated linear RNAs^1^. The advent of high-throughput sequencing technologies has enabled the identification of circRNAs with biological functions in diseases, particularly cancers. Because their stable circular structures resist digestion by RNase R, circRNAs accumulate in the brain with aging in a conservative form wherein neurons seldom undergo mitosis; some of these RNAs have been reported to serve as prognostic biomarkers for central nervous system (CNS) disease^2^. CircRNAs were initially believed not to be translated into proteins but to function as microRNA sponges or transcriptional regulators in cells. According to their origin of cyclization, circRNAs can be divided into 3 groups: exonic circRNAs (EcRNAs), intronic RNAs (CiRNAs), and exon-intron circRNAs (EIcRNAs)^3^. CiRNAs and EIcRNAs usually exist in the nucleus, whereas EcRNAs are exported into the cytoplasm^4^. Recent studies on circRNAs have shown that some EcRNAs with a short open reading frame (sORF) might encode functional proteins through 5´cap independent translation^5^. More novel proteins encoded by circRNAs have been demonstrated to play key roles in tumorigenesis, particularly that of glioblastoma multiforme (GBM). Our team has previously explored the roles of circRNA-encoded proteins in GBM; here, we comprehensively summarize their regulatory effects in GBM.

## Cap-independent translation mechanism of circRNAs

**Figure 1:**
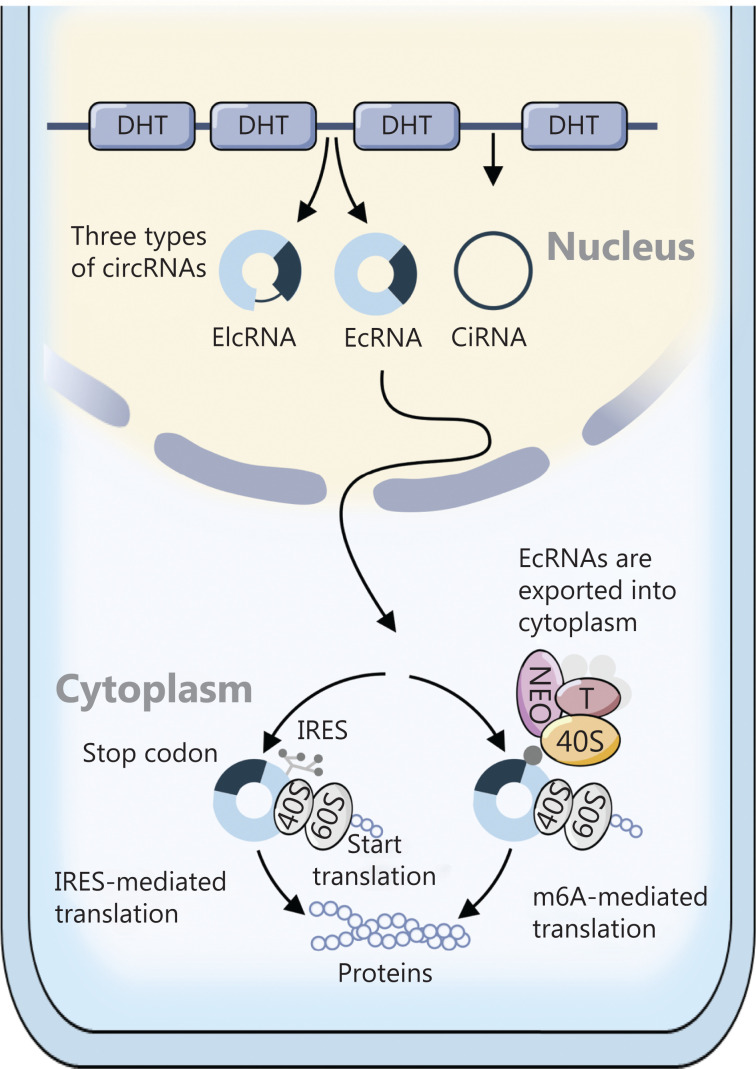
Translation mode of circRNAs in eukaryotic cells. EcRNAs are exported to the cytoplasm and then recruit 40S through their own structure. An IRES or m6A might mediate cap-independent translation.

mRNA translation in eukaryotes is usually initiated from a 7-methylguanosine cap added to the 5´ end of mRNA during its synthesis, in a process characterized as canonical translation. Non-canonical translation is cap-independent and occurs under conditions such as cellular stress or viral infection (**Figure 1**). The internal ribosome entry site (IRES), a sequence located in the 5´UTR of the mRNA, directly recruits ribosomes for translation initiation^6^. It was first found in viruses and then was widely identified in eukaryote cells. Fragments in circRNAs with AU-rich motifs (~10 nt) possess IRES-like activity and initiate translation^7^. Another translation mode depends on the methylation of adenosine residues in the 5´UTRs of RNAs. The N6-methyladenosines (m6A) in the 5´UTR bind eukaryotic initiation factor 3 (eIF3) and recruit the 43S complex, thereby initiating translation according to the 7-methylguanosine cap requirement^8^. Unlike the IRES mode, m6A translation initiation is dependent on several external factors: the process requires m6A readers, such as the YTHDF protein family, and is regulated by FTO (m6A demethylases) and METTL3/14 (m6A methyltransferases)^9^. Both IRES and m6A are widely found in circRNAs, and their translation mediated by these 2 modes has attracted substantial attention in recent years.

## Methods to identify the coding potential of circRNAs

Bioinformatics has been used to identify the coding potential of circRNAs (summarized in **Table 1**). ORF Finder is often used to identify all ORFs in RNA sequences^10^. Identification of IRES and m6A is currently available through most bioinformatic analysis websites, given the growing interest in the coding potential of circRNAs. Through integration of multiple bioinformatic studies, circRNAs containing ORFs, IRES, or m6A sequences can be screened, then experimentally validated. A dual luciferase reporting assay is subsequently necessary to assess the bioactivity of IRES, and MeRIP-qPCR can be used to detect the methylation modification of selected RNAs. Currently, plasmids for overexpression of circRNA and the linear ORF are often transfected into 293T cells. Finally, Western blot and liquid chromatography-mass spectrometry are used to validate the coding potential of circRNAs. To further study the functions of the encoded proteins, plasmids for overexpression of mutant ORF, IRES, or m6A can be used to assess whether the biological function is associated with the protein or structure of circRNAs.

**Table 1:** Bioinformatics tools for identifying the coding potential of circular RNAs

Name	Function
Circbase	Obtain the base sequence of circRNA
ORF Finder	Find possible ORFs in the selected sequence and predict amino acid sequence
IRES finder	Search for IRES in the selected sequence
CircRNADB	Integrated tool: search for ORF and IRES in circRNA
CircPro	Integrated tool: identify circRNAs with coding potential
CircCode	Integrated tool: identify circRNAs with coding potential
CircBase	Integrated tool: search for ORF, IRES, and m6A modifications in circRNA
Pfam	Search homology of the selected amino acid sequence

## CircRNA translation in GBM

Here, we describe several novel proteins encoded by circRNAs in GBM, including their biological functions and molecular mechanisms (**Table 2**).

**Table 2:** Proteins encoded by circRNAs in GBM

Protein	Gene	Phenotype and function in GBM
SHPRH-146aa	*circSHPRH*	Downregulated in GBM; alleviates malignancy *in vitro* and *in vivo*
AKT3-174aa	*circAKT3*	Downregulated in GBM; decreases cell proliferation, radiation resistance, and *in vivo* tumorigenicity
FBXW7-185aa	*circFBXW7*	Downregulated in GBM; inhibits proliferation and cell cycle acceleration *in vitro* and *in vivo*
PINT87aa	*circLINC-PINT*	Downregulated in GBM; suppresses cell proliferation *in vitro* and *in vivo*
rtEGFR	*circEGFR*	Upregulated in GBM; enhances tumorigenicity *in vitro* and *in vivo*
SMO-193aa	*circSMO*	Upregulated in GBM; enhances self-renewal and proliferation of GBM cells *in vitro*, and tumorigenicity *in vivo*
C-E-Cad	*circ-E-CAD*	Upregulated in GBM; promotes glioma stem cell tumorigenicity

## SHPRH-146aa encoded by *circSHPRH* suppresses tumorigenicity in GBM

*circSHPRH*, cyclized from the exon of *SNF2* histone linker PHD RING helicase (*SHPRH*) gene, has the above-mentioned characteristics for protein translation^11^. The UGAUGA motif has been documented in the circular base sequence of *circSHPRH*, which contains both the initiation and termination codons and an sORF. With IRES initiation, *circSHPRH* encodes a novel 146-amino-acid protein (SHPRH-146aa). SHPRH-146aa shares an overlapping amino acid sequence with the SHPRH protein and prevents the full-length SHPRH protein from being degraded by the ubiquitin-proteasome system. SHPRH-146aa has been shown to be downregulated in brains with GBM, as compared with normal brains, and to act as a tumor suppressor inhibiting the tumorigenicity and proliferation of GBM.

## AKT3-174aa encoded by *circAKT3* suppresses tumorigenicity in GBM

*CircAKT3* is cyclized from the exons of the *AKT3* gene and downregulated in GBM tissues. With IRES activity and the overlapping start-stop codon UAAUGA, *circAKT3* encodes a novel 174 amino acid protein AKT-174aa, which has a tumor-suppressor role in GBM^12^. Importantly, AKT3-174aa functions as a protein decoy that limits the phosphorylation of AKT3-Thr308 by interacting with PDK1.

## FBXW7-185aa encoded by *circFBXW7* suppresses tumorigenesis in GBM

FBXW7 is a well-characterized E3 ligase with a tumor-suppressor role. *CircFBXW7*, the circular form of the *FBXW7* gene, encodes a novel 185-amino-acid protein (FBXW7-185aa)^13^. FBXW7-185aa also acts as a protein decoy that competitively binds USP28 and subsequently prevents its binding to FBXW7α. FBXW7α, the most abundant isoform of FBXW7, suppresses tumorigenesis by inducing ubiquitination-induced degradation of c-Myc, whereas the de-ubiquitinating enzyme USP28 may stabilize c-Myc. Thus, FBXW7-185aa and *circFBXW7* are downregulated in GBM and suppress GBM tumorigenesis by enhancing the activity of FBXW7α. Similar conclusions have been drawn from a study on the role of *circFBXW7* in TNBC^14^.

## PINT87aa encoded by *circLINC-PINT* suppresses tumorigenicity in GBM

A study by Zhang et al.^15^ has indicated that the circular form of the long non-coding RNA *LINC-PINT* has translation properties and encodes a new functional protein, PINT87aa. PINT87aa has been shown to be split into three smaller fragments, then co-localized in the nucleus with the PAF1 complex, which regulates the elongation of multiple oncogenes, including *CPEB1*, *SOX-2*, and *c-Myc*. Moreover, PINT87aa anchors the PAF1 complex on the target oncogene’s promoter, thus pausing Pol II-induced mRNA elongation, and consequently inhibiting GBM proliferation and tumorigenesis. In addition, PINT87aa has been found to induce cellular senescence in hepatocellular carcinoma^16^.

## Rolling-translated EGFR (rtEGFR) encoded by *circEGFR* promotes tumorigenicity in GBM

*CircEGFR* originates from exons 14 and 15 of *EGFR* and is highly expressed in GBM, consistent with its host genes. The sORF of *circEGFR* starts with ATG but has no stop codon. Thus, the circRNA translates an non-terminating protein with an infinite ORF consisting of a repeated amino acid sequence, termed rtEGFR^17^. rtEGFR bands of 35 kD, 40 kD, 55 kD, and 70 kD have been found through Western blot. As a polymetric protein complex, rtEGFR promotes the tumorigenicity of GBM by maintaining EGFR membrane localization and attenuating the consumption of EGFR.

## Smoothened (SMO)-193aa encoded by *circSMO* promotes tumorigenicity in GBM

G protein-coupled-like receptor SMO is a core component of the Hedgehog signaling pathway, which is aberrantly activated in GBM. The release of SMO from patched transmembrane receptors (PCTH) after stimulation by Shh is the key step in activation of the Hedgehog pathway. Inhibition of SMO has been shown to be effective in some glioma cell lines with abnormal Hedgehog signaling. *circSMO* reportedly originates from exons 3–6 of *SMO* and encodes SMO-193aa, whose translation is driven by an IRES^18^. Interestingly, SMO-193aa directly interacts with SMO and enhances the cholesterol modification required for SMO release. Shh stimulation also upregulates the expression of SMO-193aa. Thus, Shh/Gli1/FUS/SMO-193aa induces a positive feedback mechanism that sustains Hedgehog signaling activation, and consequently promotes self-renewal and tumorigenesis of GBM.

## C-E-Cad encoded by *circ-E-Cad* promotes tumorigenicity in GBM

*Circ-E-Cad* is a 733-nucleotide circRNA that originates from *CDH1* and is highly expressed in GBM. *Circ-E-Cad* contains an IRES and multiple-round ORF because the stop codon TGA is not in the first round read. C-E-Cad is a 254-amino-acid protein that is translated by *circ-E-Cad* and contains a unique 14-amino acid tail at the C terminus formed by a natural frameshift in the second-round translation^18^. C-E-Cad is secreted by cells and subsequently activates EGFR independently with its 14-amino acid tail. *In vitro* and *in vivo* assays have indicated that C-E-Cad promotes GBM tumorigenicity. The 14-aa tail of C-E-Cad has been shown to be required for the EGFR-activating ability, thus providing an exceptional anti-glioma therapy target.

## Discussion and perspectives: do novel proteins have value in GBM targeting therapy?

CircRNAs are abundantly expressed in the brain and have been demonstrated to regulate CNS diseases, particularly brain cancers such as GBM. CircRNAs are involved in multiple cellular processes, including proliferation, invasion, apoptosis, and angiogenesis. Given their tissue-specific expression and highly conserved sequences, circRNAs have promising prospects as biomarkers for the diagnosis and prognosis of GBMs. CircRNAs have been reported to mediate cellular processes by sponging miRNAs, interacting with proteins, regulating gene splicing or transcription, and encoding functional proteins, among other functions. In recent years, numerous circRNAs relevant to GBMs have been identified and have provided novel insights into individualized therapy for GBM. However, circRNAs have still not been applied clinically for GBM management, possibly because of their disadvantages with respect to coding RNAs. Using coding RNAs to design targeted therapy is an attractive option that could be applied in clinical practice.

Glioma is the most common type of brain cancer, and more than half of glioma cases are GBM. The conventional therapy for GBM, including surgical excision, radiation, and chemotherapy, has poor efficacy^19^. Advances in the molecular understanding of tumorigenicity have shed light on the treatment of most cancers with diverse targeted drugs. Unfortunately, few of these targets, including VEGF, mTOR, and EGFR, are effective in the clinical treatment of GBM^20^. Rapid recurrence, chemical resistance, and brain barriers have largely limited the efficacy of medications, including chemotherapy and gene targeting therapy. The above findings highlight the need for more in-depth molecular studies on genes and proteins in GBM.

In recent years, our research team has focused on novel proteins encoded by sORFs, including proteins translated by UTR sequences, mostly lincRNAs and circRNAs. In-depth studies on coding circRNAs have supplemented the current knowledge regarding GBM and shown promise for future clinical practice. For example, although EGFR amplification and mutation are universal in GBM and have been established as biomarkers, few medications targeting EGFR are efficacious *in vivo*. The intricacy of the EGFR activation process may be responsible for this phenomenon; therefore further studies are warranted. In our previous studies, we found that rtEGFR and C-E-Cad have pivotal roles in the activation of EGFR; consequently, we were inspired to use them as new targets in combination with EGFR-targeted therapy in GBM. Moreover, AKT3-174aa, SPHRH-146aa, FBXW7-185aa and SMO-193aa have all been documented to play important roles in their host gene signaling pathways. Given that designing chemicals or antibodies is relatively easier for protein targets than RNA targets, novel functional proteins have the potential for faster clinical application. In our studies, the efficacy of potential targeted inhibitors for proteins has already been assessed in animal experiments, and the results have been satisfactory.

Coding circRNAs belong primarily to EccircRNAs produced from the exons of their host genes. Recent studies have shown that proteins encoded by circRNAs mainly intervene in the signaling pathways of host genes, possibly because of the shared sequence between the sORFs and the host genes. However, the few available studies are insufficient to indicate the mechanisms of circRNA-encoded proteins, because many translation modes are likely to await discovery.

The translation of circRNAs is widely acknowledged to be more complex than the traditional modes of translation, thus indicating a need for further research. We have found that *circEGFR* translates rtEGFR into a protein complex, owing to the absence of a stop codon, and *Circ-E-Cad* encodes a functional 14-amino acid tail by frameshifting during the second round of translation. Moreover, frameshifting could make the sORF differ from the CDS of the host gene and theoretically encode a new protein. According to current reports, the translations of circRNAs in GBM are all driven by IRES, attributable to thelimited number of studies. Generally, most studies investigating the roles of genes in GBM involve the sequencing of differentially expressed genes. However, translation driven by m6A requires an m6A reader such as YTHDF3 in the microenvironment, whereas the expression of circRNAs might not substantially differ between tumor tissue and adjacent normal tissue. Advances in histopathological and sequencing techniques, including single-cell sequencing, may aid in understanding of the translation of circRNAs driven by m6A. Small proteins have been shown to have multiple functions in cellular processes in GBM. For example, C-E-Cad is secreted from cells and subsequently activates EGFR. Many small proteins might be secreted and function in the tumor microenvironment by altering the signaling, metabolism, and immunity of GBM cells.

## Conclusions

Studies on novel proteins encoded by circRNAs may meet the demand for more biomarkers or specific targets in the treatment of GBM. Studies on small proteins may enrich knowledge of pathogenesis and provide new targets for GBM. Further advances in screening technology of circRNAs may lead to the discovery of other circRNA-encoded proteins that could potentially be combined with current therapeutic approaches to improve patient outcomes.

## References

[1] Wilusz JE (2017). Circular RNAs: unexpected outputs of many protein-coding genes. RNA Biol.

[2] Hao Z, Hu S, Liu Z, Song W, Zhao Y, Li M (2019). Circular RNAs: functions and prospects in glioma. J Mol Neurosci.

[3] Li Z, Huang C, Bao C, Chen L, Lin M, Wang X (2015). Exon-intron circular RNAs regulate transcription in the nucleus. Nat Struct Mol Biol.

[4] Hansen TB, Jensen TI, Clausen BH, Bramsen JB, Finsen B, Damgaard CK (2013). Natural RNA circles function as efficient microRNA sponges. Nature.

[5] Lei M, Zheng G, Ning Q, Zheng J, Dong D (2020). Translation and functional roles of circular RNAs in human cancer. Mol Cancer.

[6] Fitzgerald KD, Semler BL (2009). Bridging IRES elements in mRNAs to the eukaryotic translation apparatus. Biochim Biophys Acta.

[7] Yang Y, Wang Z (2019). IRES-mediated cap-independent translation, a path leading to hidden proteome. J Mol Cell Biol.

[8] Coots RA, Liu XM, Mao Y, Dong L, Zhou J, Wan J (2017). m(6)A facilitates eIF4F-independent mRNA translation. Mol Cell.

[9] Zhang Y, Geng X, Xu J, Li Q, Hao L, Zeng Z (2021). Identification and characterization of N6-methyladenosine modification of circRNAs in glioblastoma. J Cell Mol Med.

[10] Rombel IT, Sykes KF, Rayner S, Johnston SA (2002). ORF-FINDER: a vector for high-throughput gene identification. Gene.

[11] Zhang M, Huang N, Yang X, Luo J, Yan S, Xiao F (2018). A novel protein encoded by the circular form of the SHPRH gene suppresses glioma tumorigenesis. Oncogene.

[12] Xia X, Li X, Li F, Wu X, Zhang M, Zhou H (2019). A novel tumor suppressor protein encoded by circular AKT3 RNA inhibits glioblastoma tumorigenicity by competing with active phosphoinositide-dependent Kinase-1. Mol Cancer.

[13] Yang Y, Gao X, Zhang M, Yan S, Sun C, Xiao F (2018). Novel role of FBXW7 circular RNA in repressing glioma tumorigenesis. J Natl Cancer Inst.

[14] Ye F, Gao G, Zou Y, Zheng S, Zhang L, Ou X (2019). circFBXW7 inhibits malignant progression by sponging miR-197-3p and encoding a 185-aa protein in triple-negative breast cancer. Mol Ther Nucleic Acids.

[15] Zhang M, Zhao K, Xu X, Yang Y, Yan S, Wei P (2018). A peptide encoded by circular form of LINC-PINT suppresses oncogenic transcriptional elongation in glioblastoma. Nat Commun.

[16] Xiang X, Fu Y, Zhao K, Miao R, Zhang X, Ma X (2021). Cellular senescence in hepatocellular carcinoma induced by a long non-coding RNA-encoded peptide PINT87aa by blocking FOXM1-mediated PHB2. Theranostics.

[17] Liu Y, Li Z, Zhang M, Zhou H, Wu X, Zhong J (2021). Rolling-translated EGFR variants sustain EGFR signaling and promote glioblastoma tumorigenicity. Neuro Oncol.

[18] Wu X, Xiao S, Zhang M, Yang L, Zhong J, Li B (2021). A novel protein encoded by circular SMO RNA is essential for Hedgehog signaling activation and glioblastoma tumorigenicity. Genome Biol.

[19] Stupp R, Mason WP, van den Bent MJ, Weller M, Fisher B, Taphoorn MJ (2005). Radiotherapy plus concomitant and adjuvant temozolomide for glioblastoma. N Engl J Med.

[20] Westphal M, Maire CL, Lamszus K (2017). EGFR as a target for glioblastoma treatment: an unfulfilled promise. CNS Drugs.

